# Solithromycin mitigates *Prevotella intermedia*–induced methicillin-resistant *Staphylococcus aureus* ventilator-associated pneumonia by enhancing alveolar macrophage function

**DOI:** 10.3389/fcimb.2026.1723186

**Published:** 2026-02-09

**Authors:** Koki Fukushima, Naoki Iwanaga, Nobuyuki Ashizawa, Kazuaki Takeda, Tatsuro Hirayama, Masataka Yoshida, Shotaro Ide, Takahiro Takazono, Kosuke Kosai, Noriho Sakamoto, Mariko Naito, Katsunori Yanagihara, Hiroshi Mukae

**Affiliations:** 1 Department of Respiratory Medicine, Nagasaki University Graduate School of Biomedical Sciences, Nagasaki, Japan; 2 Department of Respiratory Medicine, Nagasaki University Hospital, Nagasaki, Japan; 3 Department of Pharmacotherapeutics, Nagasaki University Graduate School of Biomedical Sciences, Nagasaki, Japan; 4 Infectious Diseases Experts Training Center, Nagasaki University Hospital, Nagasaki, Japan; 5 Department of Infectious Diseases, Nagasaki University Graduate School of Biomedical Sciences, Nagasaki, Japan; 6 Department of Laboratory Medicine, Nagasaki University Hospital, Nagasaki, Japan; 7 Department of Microbiology and Oral Infection, Nagasaki University Graduate School of Biomedical Sciences, Nagasaki, Japan

**Keywords:** alveolar macrophages, immunomodulation, methicillin-resistant *Staphylococcus aureus*, *Prevotella intermedia*, RNAsequencing, solithromycin, ventilator-associated pneumonia

## Abstract

**Background:**

Ventilator-associated pneumonia (VAP) is a fatal intensive care infection. VAP caused by methicillin-resistant *Staphylococcus aureus* (MRSA) can be exacerbated by *Prevotella intermedia* culture supernatant (*P. int.* sup.). Solithromycin (SOL), a fourth-generation macrolide, inhibits bacterial protein synthesis and modulates immunity; however, its effects on exacerbation of MRSA-VAP by *P. int*. sup. remain unclear. This study examined whether SOL inhibits bacterial protein synthesis by binding to the 50S ribosomal subunits in *P. int*. sup. and subsequently reduces the worsening of MRSA-VAP caused by *P. int.* sup.

**Methods:**

BALB/cCrSlc mice received MRSA and *P. int.* sup. with or without sub-minimum inhibitory concentrations of SOL (*P. int*. sup. (SOL)) or clarithromycin (CAM; *P. int.* sup. (CAM)). Outcomes included survival rates, lung MRSA burden, and transcriptomics (reverse transcription polymerase chain reaction, bulk RNA sequencing [RNA-seq]). *In vitro*, bone marrow-derived alveolar macrophage-like cells (AMLCs) from C57BL/6J mice were infected with MRSA ± SOL; bactericidal activity and mRNA expression were measured.

**Results:**

*P. int*. sup. increased mortality, bacterial load, and neutrophilic infiltration; however, *P. int*. sup. (SOL) significantly improved survival rate (100%, *n* = 8, ****P < 0.0001), reduced MRSA burden (*n* = 10–11, **P < 0.01), and enhanced macrophage recruitment (*n* = 7–8, ****P < 0.001). *P. int.* sup. downregulated *Ccr2* expression (*n* = 7–8, ***P < 0.001). RNA-seq analysis revealed *P. int*. sup. (SOL) upregulated macrophage phagocytosis and bactericidal pathways. SOL-pretreated AMLCs infected with MRSA exhibited reduced bacterial burden (*n* = 8, *P < 0.05 vs control, **P < 0.01 vs CAM-pretreated AMLCs) and upregulated *Tnf-α* expression (*n* = 7–8, *P < 0.05 vs control).

**Conclusion:**

SOL protects by activating alveolar macrophages and promoting TNF-related responses, suggesting a novel immunomodulatory role for SOL in host defense against exacerbation of MRSA-VAP by *P. int.* sup.

## 1 Introduction

Ventilator-associated pneumonia (VAP) is one of the most common infectious complications in intensive care units, occurring in approximately 9–27% of intubated patients, and is associated with high mortality rates (American Thoracic SocietyInfectious Diseases Society of America, 2005, Papazian et al., 2020; Mukae et al., 2025). A systematic review of VAP cases in Japan reported *Pseudomonas aeruginosa* (29.2%) and methicillin-resistant *Staphylococcus aureus* (MRSA; 12.0%) as the predominant pathogens (Moro et al., 2024). In contrast, 16S rRNA gene analyses have shown that anaerobic and oral bacteria are more frequently isolated from patients with community-acquired pneumonia (Yamasaki et al., 2013; Nemoto et al., 2022). *Prevotella intermedia* is an obligate anaerobe residing in periodontal pockets and is a causative agent of periodontal disease (Kesavalu et al., 2007; Mohanty et al., 2019). Notably, several studies have demonstrated that poor oral hygiene can predispose individuals to respiratory infections (Sjögren et al., 2008; Hata et al., 2020). A previous study showed that treatment with *P. intermedia* culture supernatant (*P. int.* sup.) exacerbated pneumonia caused by oral streptococci, *Streptococcus pneumoniae*, and MRSA in mouse models (Nagaoka et al., 2014; Yamashita et al., 2020; Ashizawa et al., 2025), whereas culture supernatants from *Fusobacterium nucleatum* or *Porphyromonas gingivalis* did not worsen the disease (Nagaoka et al., 2014), suggesting a specific virulence-promoting effect of *P. int.* sup. *In vitro*, *P. int*. sup. has also been shown to upregulate *CXCL8* gene expression and interleukin (IL)-8) secretion in human airway epithelial cells, responses that were significantly suppressed by pretreatment with clarithromycin (CAM) (Iwanaga et al., 2024).

Solithromycin (SOL) is a fourth-generation macrolide antibiotic, structurally related to telithromycin, that inhibits bacterial protein synthesis by binding to the 50S ribosomal subunit (Fernandes et al., 2016). Macrolides have been reported to modulate immune responses, in addition to their antimicrobial effects (Kadota et al., 1993; Mukae et al., 1995; Kobayashi et al., 2013). They can also inhibit pneumolysin production which is a major pathogenic factor of *S. pneumonia* at sub-minimum inhibitory concentrations (Fukuda et al., 2006), However, it remains unclear whether SOL can suppress the pathogenic exacerbation of MRSA-VAP by *P. int*. sup.

Therefore, in this study, we investigated the protective efficacy of SOL against *P. int*. sup.-mediated exacerbation of MRSA-VAP and explored its underlying mechanisms both *in vivo* and *in vitro*, with a focus on immune cell modulation, particularly alveolar macrophages.

## 2 Materials and methods

### 2.1 Mice

Male BALB/cCrSlc mice (7–8 weeks old; wild-type [WT]; Japan SLC, Japan) were used to establish VAP mice model which requires technically demanding airway administration (e.g., intratracheal/oropharyngeal delivery) *in vivo*, and female C57BL/6J mice (7–8 weeks old; WT; KBT ORIENTAL Co., Ltd., Japan) were used *in vitro* to make AMLCs followed the previous method (as below section 2.4). Both mice maintained under specific pathogen-free conditions at the Research Center for Biomedical Models and Animal Welfare, Nagasaki University Graduate School of Biomedical Sciences. This study was approved by Nagasaki University (approval no. 2109091745), All animal experiments were conducted in accordance with the Animal Research: Reporting of *In Vivo* Experiments (ARRIVE) guidelines and the regulations of the Research Center for Biomedical Models and Animal Welfare at Nagasaki University. No unexpected adverse events occurred during the study period. This study did not apply humane endpoints, randomization, measures to minimize confounders, or inclusion criteria to the animals or data. Our study exclusively used female mice because the mice MRSA-VAP model required larger individuals.

### 2.2 SOL, CAM, *P. int.* sup. (SOL), and *P. int.* sup. (CAM)

SOL was a gift from FUJIFILM Toyama Chemical Co., Ltd., and CAM, widely used in macrolide antibiotic was purchased from Sigma-Aldrich, Japan as a control group. Both antibiotics were dissolved in dimethyl sulfoxide (DMSO; FUJIFILM Toyama Chemical Co., Ltd., Japan) and stored at −30°C. SOL and CAM were added to the Gifu anaerobic medium (GAM) broth (Shimadzu Diagnostics Co., Japan) at sub-minimum inhibitory concentrations (sub-MIC) that permitted growth of *P. int.* (0.00019 μg/mL for SOL and 0.0039 μg/mL for CAM), under the same incubation conditions used for culturing *P. intermedia* in GAM broth.

### 2.3 Bacteria

The MRSA clinical isolate NU 101 was cultured on tryptic soy agar (TSA) II 5% Sheep Blood Agar M (Becton Dickinson and Company) for 24 h at 37°C with 5% CO_2_. The bacteria were then resuspended in TS broth (TSB; Becton Dickinson and Company) and incubated at 37°C with shaking for 4 h to reach the logarithmic phase of MRSA growth. *P. intermedia* strain OMA14 was isolated from periodontal pockets of a Japanese patient with periodontitis (Naito et al., 2022). *P. int.* was cultured on PV Brucella HK agar (Kyokuto Pharmaceutical Industrial Co., Japan) for 48 h under anaerobic conditions, then scraped and suspended in modified GAM broth (Shimadzu Diagnostics Co., Japan). To prepare *P. int.* sup., *P. intermedia* was incubated in GAM broth in an anaerobic chamber for 36–48 h with or without SOL or CAM at the minimum concentrations that allowed bacterial growth. After incubation, bacterial suspensions were centrifuged (1, 690 x *g* for 10 min), supernatants collected, filtered through a 0.22-μm pore-size membrane filter (Sartorius Japan K.K., Japan), and sterility was confirmed by the absence of microbial growth both on PV Brucella HK agar, counting CFU, and in GAM broth, measuring OD at 600nm. To determine which active components in *P. int.* sup. were responsible for the observed effects, heat-treated *P. int.* sup. (56°C, 30 min) was also prepared. The 90% MIC (MIC_90_) of both SOL and CAM was 32 μg/mL.

### 2.4 Alveolar macrophage-like cells

Generation of AMLCs from undifferentiated bone marrow (BM) cells *in vitro* was conducted following a method previously described (Luo et al., 2021). Briefly, BM cells were harvested from 7- to 8-week-old female C57BL/6J (WT) mice and cultured in 12-well plates in Dulbecco’s modified Eagle medium (DMEM; Thermo Fisher Scientific, Japan) supplemented with 10% heat-inactivated FBS (Sigma-Aldrich, Japan), penicillin (Sigma-Aldrich), and streptomycin (Sigma-Aldrich). The cultures were maintained for 7 d in the presence of granulocyte-macrophage colony-stimulating factor (GM-CSF; Funakoshi Company, Japan) and transforming growth factor beta (TGF-β; Funakoshi Company), followed by the addition of the peroxisome proliferator-activated receptor gamma agonist rosiglitazone (Sigma-Aldrich), for a total culture period of 9 d. The differentiated AMLCs were identified using flow cytometry based on surface marker expression: Siglec-F^high^, CD11c^high^, F4/80^high^, and CD11b^low^ (49.4% of the total cell count). The AMLC phenotype was further assessed by flow cytometry based on surface marker expression. Specifically, AMLCs that are both CD80^low^ and CD163^low^ were identified as non-polarized macrophages (99.6% of the total cell count). The viability of AMLCs was confirmed in more than 90% of the total cell count using Acridine Orange/Propidium Iodide staining (Biosystems, Barcelona, Spain) and the LUNA-FL Dual Fluorescence Cell Counter (Biosystems). AMLCs were stored at 1.0 × 10^6^ cells/mL in Cell Banker (Zenogen Pharma, Japan) at −80°C or in liquid nitrogen.

### 2.5 Infection

MRSA stocks stored at −80°C were thawed and cultured on TSA II 5% Sheep Blood Agar M for 24 h at 37°C with 5% CO_2_. The bacteria were then resuspended in TSB and incubated at 37°C with shaking for 4 h to reach the logarithmic phase of growth. The bacterial suspension was centrifuged (1, 690 x *g* for 10 min), and the pellets were resuspended in phosphate-buffered saline (PBS). Bacterial concentrations were adjusted using the McFarland standard. For the VAP model, following a previous study (Yanagihara et al., 1997), mice were intubated with a 3 mm feeding tube (Atom Corporation) and oropharyngeally administered 50 μL of *P. int*. sup. *P. int.* sup. (SOL), *P. int.* sup. (CAM), or GAM broth alone, mixed with 50 μL of MRSA suspension (1.0 × 10^8^ colony-forming unit [CFU]/mL) under intraperitoneal anesthesia with midazolam, medetomidine, and butorphanol. Atipamezole was administered post-challenge. Protein concentrations in *P. int*. sup., *P. int.* sup. (SOL), *P. int.* sup. (CAM), and GAM broth were measured using a bicinchoninic acid (BCA) Protein Assay Kit (Thermo Fisher Scientific) and adjusted to equal levels. *In vitro*, AMLCs (1.0 × 10^5^ cells/well) were seeded in 24-well plates and infected with MRSA (1.0 × 10^6^ or 1.0 × 10^5^ CFU/well). In treatment experiments, *in vivo*, interferon (IFN)-β1 (BioLegend, Japan) or IFN-γ (PeproTech, Inc., USA) was oropharyngeally administered at a dose of 1 μg per mouse (50 μL) 1 h prior to MRSA infection under anesthesia with inhaled isoflurane. Protein concentrations *in P. int.* sup., *P. int.* sup. (SOL), *P. int.* sup. (CAM), and GAM broth were measured using the BCA Protein Assay Kit and quantified at 562 nm using the Multiskan SkyHigh plate reader (Thermo Fisher Scientific, Japan). The samples were then adjusted to contain the same protein concentration of 8 mg/mL. Furthermore, SOL was oropharyngeally administered at a dose of 0.00019 μg or 0.019 μg per mouse (100 μL) without MRSA infection under anesthesia with inhaled isoflurane. *In vitro*, AMLCs (1.0 × 10^5^ cells/well) were seeded in 24-well plates and applied SOL (0.00019 μg/mL) or CAM (0.0039 μg/mL)) without MRSA.

### 2.6 Bacterial burden quantification *in vivo*

Mice from each group were euthanized *via* CO_2_ exposure and exsanguinated 24 h after infection. The left lung was collected throughout all experiments to avoid inter-lobar variability (Alma et al., 2023), homogenized in 1 mL PBS, and plated on Luria–Bertani (LB) agar (Formedium Ltd., Norfolk House, UK) via serial dilution, followed by incubation at 37°C, 5% CO_2_ for 24 h. The bacterial burden was quantified based on CFU counts. Uniform distribution of the inoculum in both lungs was confirmed using trypan blue solution (Thermo Fisher Scientific, Japan).”

### 2.7 Bacterial growth assay

MRSA cultures were standardized to an optical density at 600 nm (OD_600_) of 0.1, suspended in TSB, and mixed with *P. int*. sup., *P. int*. sup. (SOL), and *P. int*. sup. (CAM) at various dilutions (undiluted, 2-fold, 3-fold, and 4-fold), or with GAM broth alone, in 96-well plates. OD_600_ was measured every hour using a plate reader.

### 2.8 Bactericidal activity assay of AMLCs

AMLCs were co-incubated with SOL, CAM, or DMSO at 37°C with 5% CO_2_ for 1 or 24 h. After removing the medium and washing three times with DMEM, the cells were infected with MRSA and incubated again at 37°C with 5% CO_2_ for 24 h. The culture medium was then collected and plated onto LB agar. The plates were incubated at 37°C with 5% CO_2_, and CFU were enumerated.

### 2.9 Single cell digestion of lung tissue and flow cytometry

After euthanasia, mouse lung tissue (left lobe) was harvested and minced. Samples were incubated with 2 mg/mL collagenase (Sigma-Aldrich, Japan) and 20 U/mL DNase-1 (Sigma-Aldrich, Japan) for 60 minutes at 37°C in a cell culture incubator with gentle rotation. The digested tissue was then passed through a 70-µm cell strainer, and the resulting cell suspension was treated with ACK lysis buffer (Thermo Fisher Scientific) for 3 minutes. Cells were fixed in 4% paraformaldehyde phosphate buffer solution (FUJIFILM Toyama Chemical Co., Ltd) for 20 minutes. Samples were kept on ice until processing, then centrifuged at 400 × g for 10 minutes at 4°C. The cell pellets were resuspended in fluorescence-activated cell sorting (FACS) buffer (PBS containing 1% bovine serum albumin). Nonspecific binding was prevented using a purified rat anti-mouse antibody targeting the FcγRIII/II receptor (CD16/CD32) (BD Bioscience, USA). After blocking, the samples were washed and resuspended in FACS buffer, then incubated with appropriate antibodies (**Supplementary Table S1**). Cells were washed again in FACS buffer and analyzed using an Attune NxT Flow Cytometer (Thermo Fisher Scientific).

### 2.10 Real-time quantitative reverse transcription–polymerase chain reaction (RT-qPCR) 

*In vivo*, mice were euthanized *via* CO_2_ exposure and exsanguinated 12 h after infection. This time point was chosen because the number of viable bacteria in the lungs was nearly equal among groups, thereby minimizing bias due to differences in lung bacterial burden. RNA was extracted from a portion of lung tissue using the RNeasy Plus Mini Kit (QIAGEN K.K., Tokyo, Japan). *In vitro*, AMLCs were pretreated with SOL or CAM for 1 h, followed by infection with MRSA for 4 h. Cells were then harvested, and total RNA was extracted using the RNeasy Mini Kit (QIAGEN, Japan). cDNA was synthesized using the iScript Reverse Transcription Supermix for RT-qPCR (Bio-Rad). qPCR was performed on an Applied Biosystems QuantStudio 12K Flex system (Thermo Fisher Scientific) using TaqMan PCR Master Mix (Thermo Fisher Scientific) and premixed primer–probe sets (Thermo Fisher Scientific; **Supplementary Table S2**).

### 2.11 Bulk RNA sequencing (RNA-seq)

Total RNA from whole lung tissue was used for RNA-seq. Mice were euthanized *via* CO_2_ exposure and exsanguinated 12 h after infection. This time point was chosen for the same reason as that stated in the previous section. RNA was extracted from a portion of lung tissue using the RNeasy Plus Mini Kit (QIAGEN K.K., Tokyo, Japan). cDNA was synthesized as previously described. RNA-seq library preparation, sequencing, read mapping, gene expression analysis, and Gene Ontology enrichment analyses were performed using DNAFORM software (Yokohama, Kanagawa, Japan). Double-stranded cDNA libraries were prepared using the SMART-Seq Stranded Kit (Clontech, 634442) and the DNBSEQ MGIEasy Universal Library Conversion Kit (MGI Tech, 1000004155), according to the manufacturers’ instructions. The libraries were sequenced with paired-end reads (150 base pairs for both reads 1 and 2) on a DNBSEQ-G400RS instrument (MGI Technology).

### 2.12 Statistics

GraphPad Prism (version 10; GraphPad Inc., USA) was used to generate graphs and perform statistical analyses. All experiments were conducted independently in at least duplicate, and data are presented as the mean ± standard error of the mean. Survival differences were analyzed using the log-rank test with Kaplan–Meier survival curves. A P-value of < 0.05 was considered statistically significant. Comparisons between two groups were performed using the non-parametric Mann–Whitney *U*-test, while comparisons among more than two groups were analyzed using either one-way analysis of variance followed by Tukey’s multiple comparisons test (for parametric data) or the Kruskal–Wallis test followed by Dunn’s multiple comparisons test (for non-parametric data).

## 3 Results

### 3.1 *P. int.* sup. reduced survival and increased lung bacterial load in MRSA-VAP mice, effects attenuated by SOL-, CAM-, or heat-treated *P. int.* sup.

**Figure 1:**
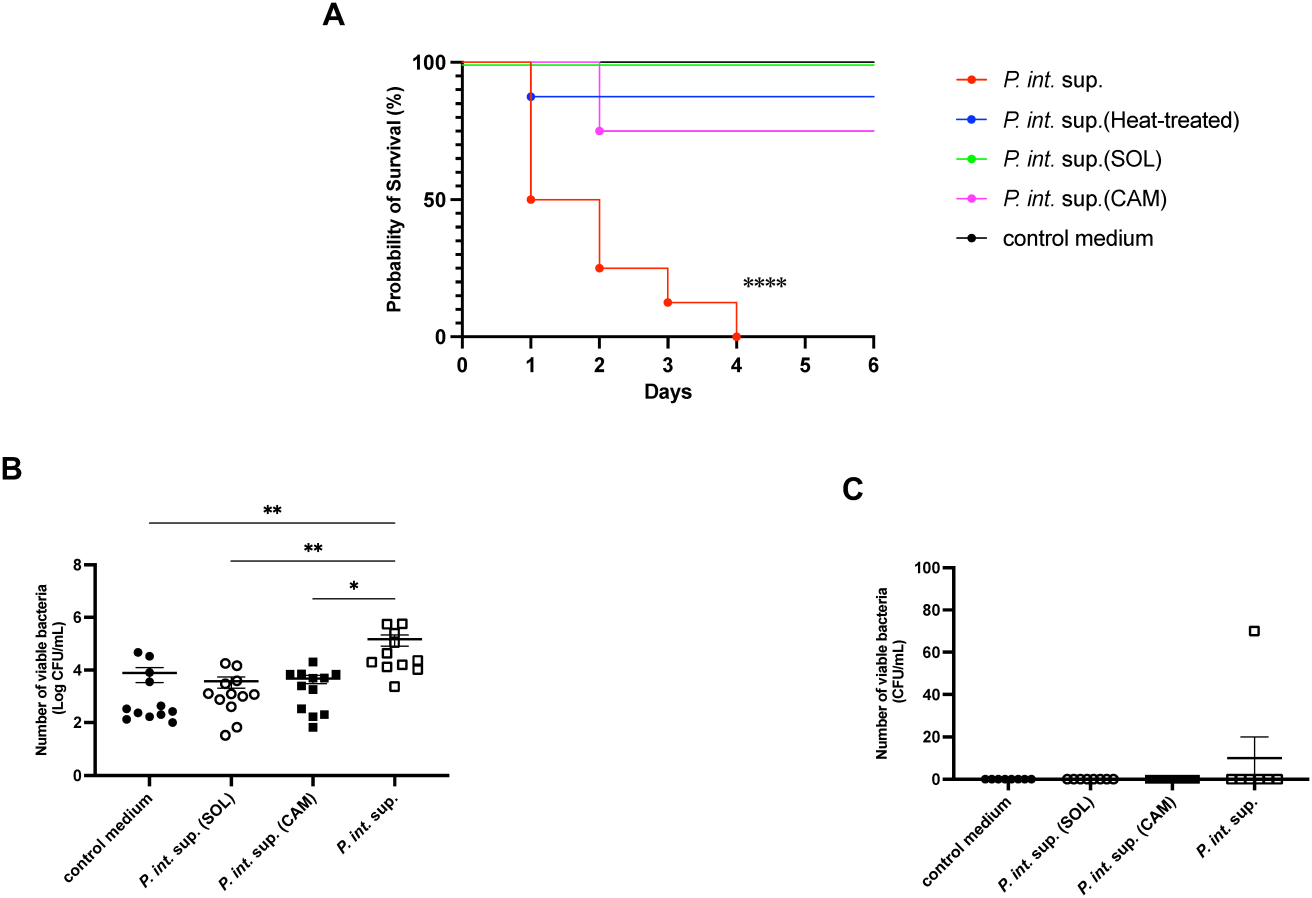
Effect of *Prevotella intermedia* culture supernatant (*P. int.* sup.) on mouse survival and lung methicillin-resistant *Staphylococcus aureus* (MRSA) burden. **(A)** Survival of BALB/cCrSlc mice infected with MRSA (5.0 × 10^5^ colony-forming unit [CFU] per mouse) in the presence of *P. int.* sup., heat-treated (56°C, 30 min) *P. int.* sup., solithromycin (SOL)-treated *P. int.* sup., clarithromycin (CAM)-treated *P. int.* sup., or control medium (*n* = 8; log-rank test; ****P < 0.0001). **(B)** MRSA CFU in BALB/cCrSlc mouse lungs at 24 h post-infection. (*n* = 10–11; Kruskal–Wallis test followed by Dunn’s multiple comparison test; **P < 0.01, **P < 0.01, *P < 0.05). Each experiment was conducted independently in duplicate. Graphs represent cumulative samples; results are expressed as mean ± standard error of the mean (SEM). **(C)** Bacterial loads in the spleen were minimal under all conditions at 24 h post-infection. Each experiment was conducted independently in duplicate. Graphs represent cumulative samples; results are expressed as mean ± standard error of the mean (SEM).

To clarify the pathogenic differences between *P. int.* sup. and SOL-treated *P. int*. sup. in MRSA-VAP mice, mortality and bacterial burdens in the lung and spleen were evaluated*. P. int*. sup. significantly reduced survival in MRSA-VAP mice, an effect that was attenuated by SOL-, CAM-, or heat-treated *P. int*. sup. (**Figure 1A**). *P. int*. sup. also increased bacterial burden in the lungs, which was similarly reduced by SOL- or CAM-treated *P. int*. sup. (**Figure 1B**). No significant differences in splenic bacterial load were observed across groups (**Figure 1C**). In summary, *P. int.* sup. reduced survival and increased lung bacterial load in MRSA-VAP mice, which was attenuated by SOL-treated *P. int.* sup.

### 3.2 *P. int.* sup. increases neutrophil infiltration, whereas SOL-treated *P. int.* sup. promotes macrophage recruitment in mouse lungs

**Figure 2:**
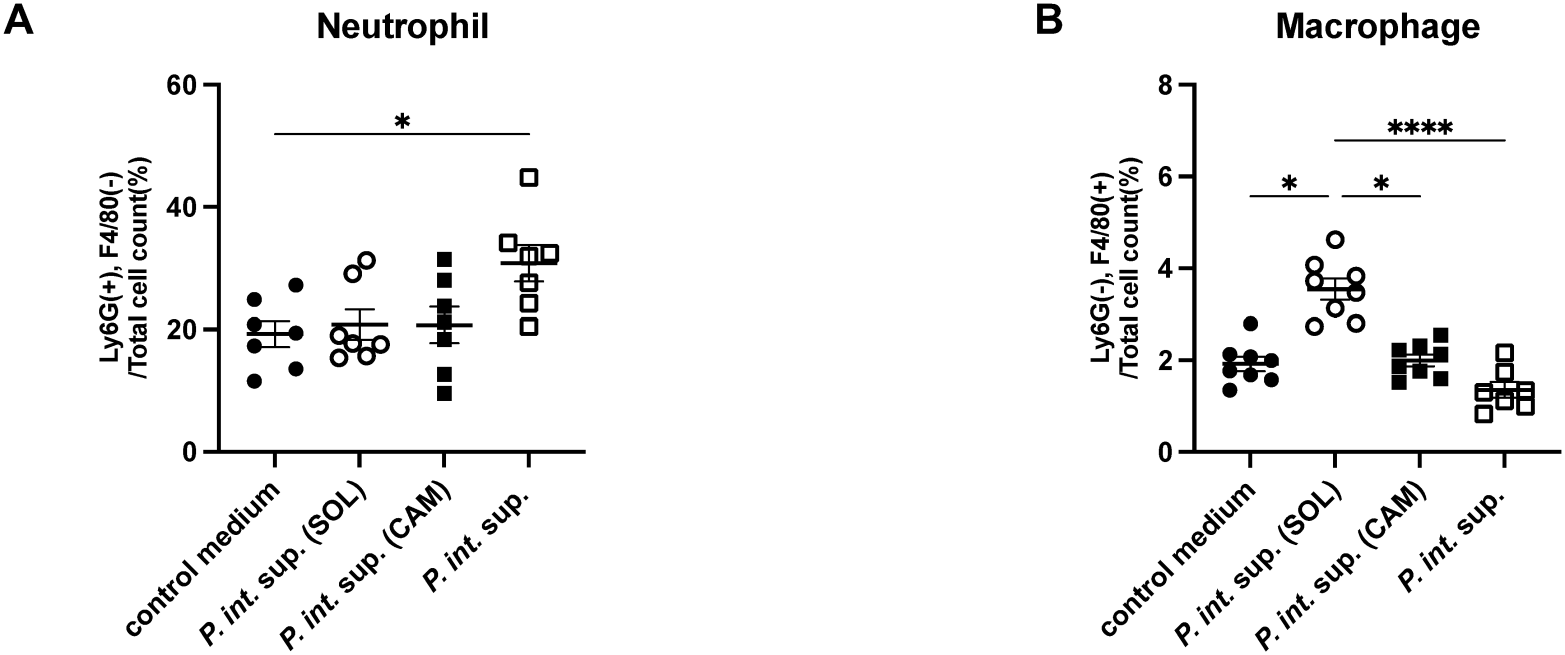
Flow cytometry analysis of cell surface antigens in BALB/cCrSlc mouse lungs 24 h post-infection with MRSA (5.0 × 10^5^ colony-forming unit [CFU] per mouse) in the presence of *P. int.* sup., solithromycin (SOL)-treated *P. int.* sup., clarithromycin (CAM)-treated *P. int.* sup., or control medium. Cells were gated based on Ly6G and F4/80 expression, and the frequency of each population was quantified as a percentage of total cells. **(A)** Staining for neutrophils (Ly6G^+^, F4/80^−^, *n* = 7–8; one-way analysis of variance (ANOVA) test followed by Tukey’s multiple comparison test; *P < 0.05). **(B)** Staining for macrophages (Ly6G^−^, F4/80^+^, *n* = 7–8; one-way ANOVA test followed by Tukey’s multiple comparison test; ****P < 0.001; ****P < 0.001, ****P < 0.001). Each experiment was conducted independently in duplicate. Graphs represent cumulative samples; results are expressed as mean ± SEM.

For identifying the immune cell populations responsible for the pathogenicity of *P. int*. sup., flow cytometric analysis was performed. In mouse lungs 24 h after MRSA infection in the presence of *P. int.* sup., *P. int.* sup. (SOL), or *P. int.* sup. (CAM), neutrophils (Ly6G^+^, F4/80^−^) were more abundant in the *P. int.* sup. group than in the control medium group. This increase was not observed in the *P. int.* sup. (SOL) or *P. int.* sup. (CAM) (**Figure 2A**). In contrast, macrophages (Ly6G^−^, F4/80^+^) were most abundant in the *P. int.* sup. (SOL) group and least abundant in the *P. int.* sup. (**Figure 2B**). No significant differences were observed in other cell types, including B cells, CD4^+^ T cells, CD8^+^ T cells, γδ cells, and NK cells (**Supplementary Figure S1**). Considering the results, SOL-treated *P. int.* sup. promotes macrophage recruitment in mouse lungs.

### 3.3 *P. int.* sup. upregulates *Ly6G* and downregulates *Ccr2* mRNA expression, whereas SOL-treated *P. int.* sup. upregulates *Tnf-α* and *Ifn-γ* mRNA expression in MRSA-VAP mice

**Figure 3:**
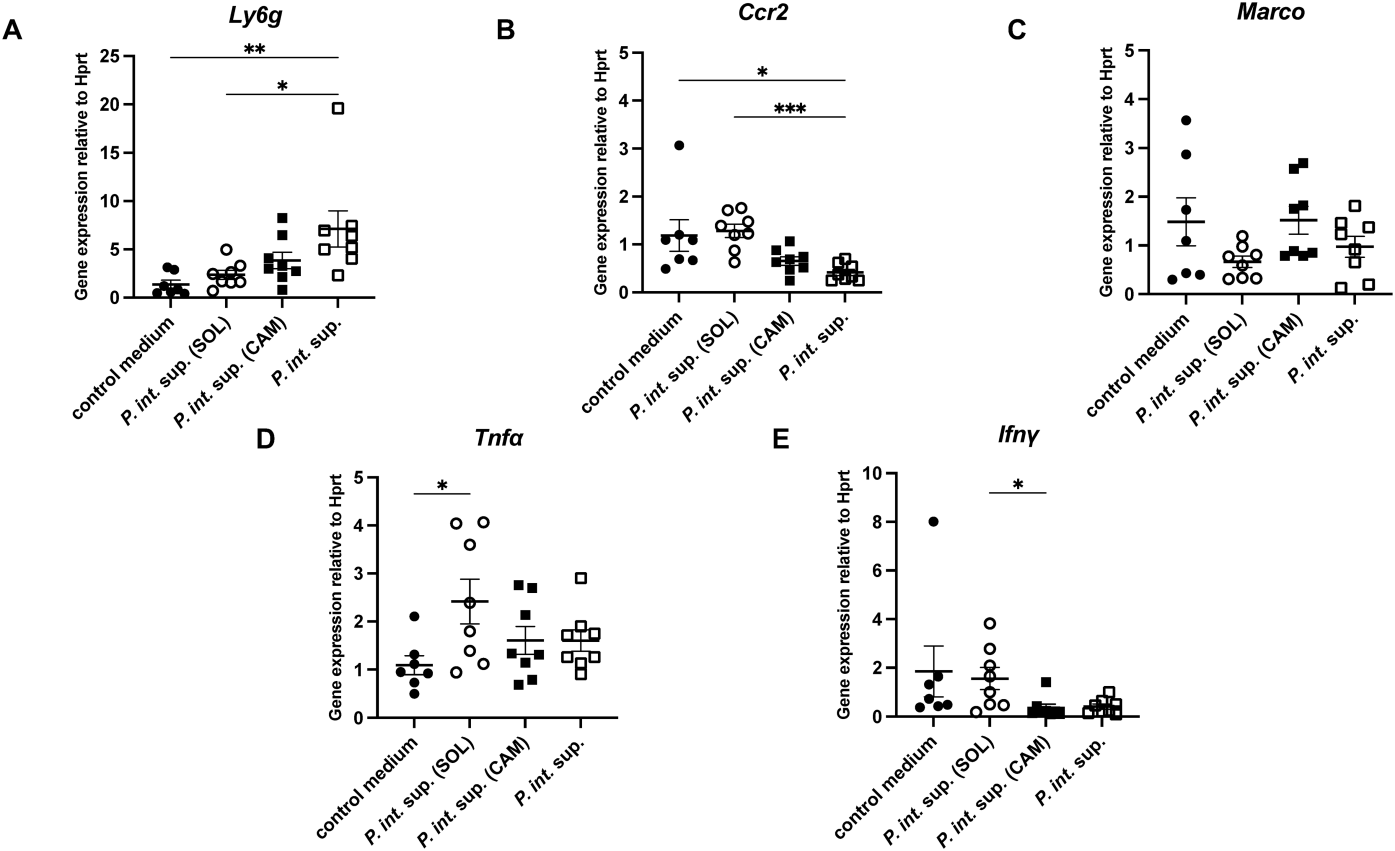
mRNA expression in BALB/cCrSlc mouse lungs 24 h post-infection with MRSA (5.0 × 10^5^ colony-forming unit [CFU] per mouse) in the presence of *P. int.* sup., solithromycin (SOL)-treated *P. int.* sup., or control medium. **(A)***Ly6G* mRNA expression was shown (*n* = 7–8; one-way ANOVA test followed by Tukey’s multiple comparison test; **P < 0.01, *P < 0.05). **(B)***Ccr2* mRNA expression was shown (*n* = 7–8; Kruskal–Wallis test followed by Dunn’s multiple comparison test; *P < 0.05, ***P < 0.001). **(C)***Marco* mRNA expression was shown(*n* = 7–8; Kruskal–Wallis test followed by Dunn’s multiple comparison test). **(D)***Tnfα* mRNA expression was shown (*n* = 7–8; one-way ANOVA test followed by Tukey’s multiple comparison test; *P < 0.05). **(E)** Interferon (*Ifn*)*-γ* mRNA expression was shown (*n* = 7–8; Kruskal–Wallis test followed by Dunn’s multiple comparison test; *P < 0.05). Gene expression levels were normalized to *Hprt* as an internal control. Each experiment was independently conducted in duplicate. Graphs represent the cumulative samples; results are expressed as mean ± SEM.

To investigate macrophage and neutrophil-associated immune functions, we quantified mRNA expression of cytokines and macrophage polarization markers. In mouse lungs 12 h after MRSA infection in the presence of *P. int.* sup., *P. int.* sup. (SOL), or *P. int.* sup. (CAM), a significant upregulation of *Ly6G* expression in the *P. int.* sup. group compared to that in the control and *P. int.* sup. (SOL) groups (**Figure 3A**), and a significant downregulation of *Ccr2* mRNA expression in the *P. int.* sup. group compared to that in the same groups (**Figure 3B**). Expression of macrophage receptor with collagenous structure (*Marco*) in the *P. int.* sup. (SOL) group was downregulated compared to that in the *P. int.* sup. (CAM) (**Figure 3C**). Additionally, *Tnf-α* expression was upregulated in the *P. int.* sup. (SOL) group compared to that in the control medium group (**Figure 3D**), and *Ifn-γ* was higher in the *P. int.* sup. (SOL) group than that in the *P. int.* sup. (CAM) group (**Figure 3E**). Based on the results presented, *P. int*. sup. downregulates *Ccr2* expression; in contrast, SOL-treated *P. int*. sup. upregulates *Ccr2* and *Tnf-α* expression in MRSA-VAP mice.

### 3.4 *P. int.* sup. upregulates the expression of inflammatory genes, whereas SOL-treated *P. int.* sup. downregulates them but upregulates genes associated with antimicrobial function in macrophages

**Figure 4:**
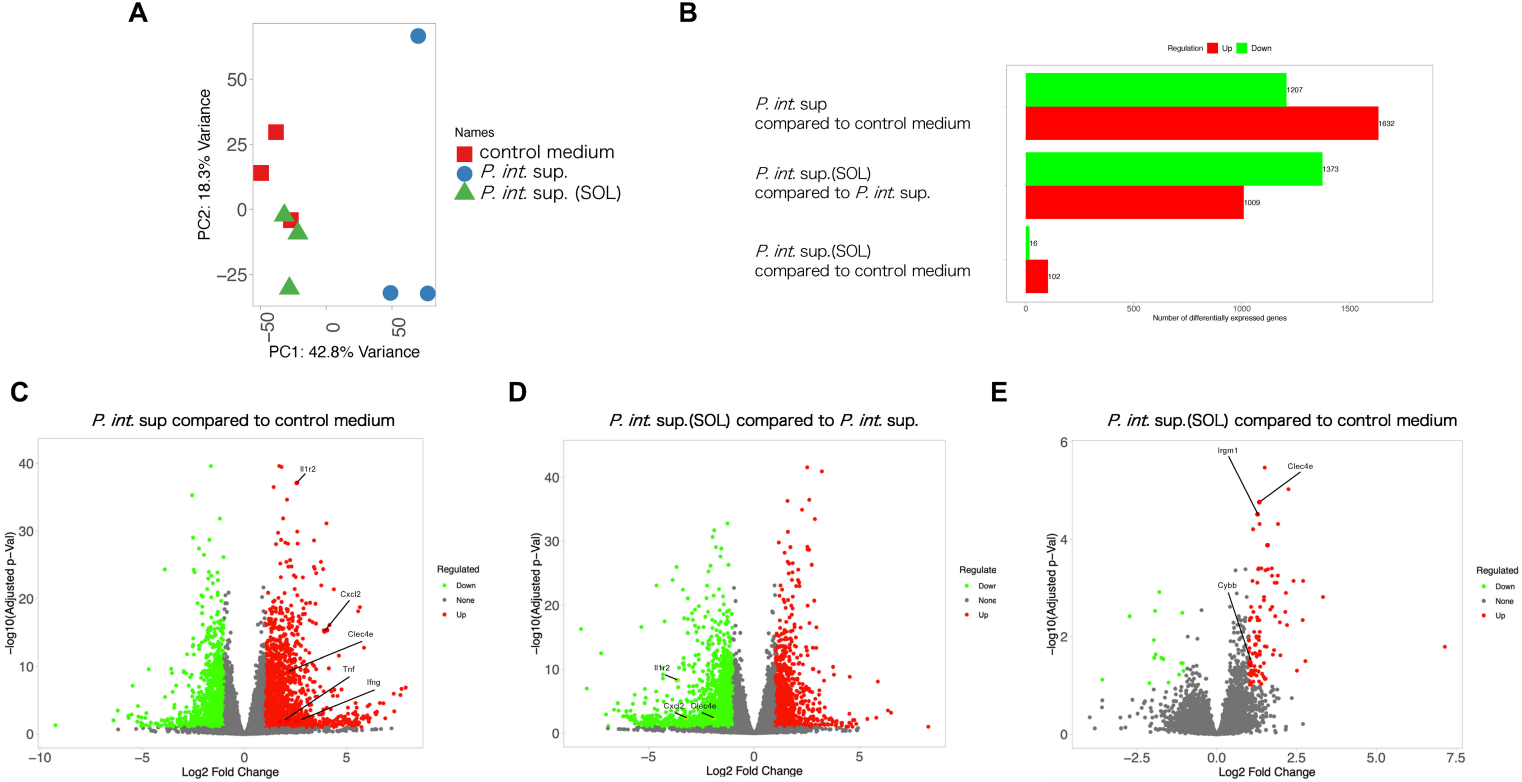
Bulk RNA sequencing (RNA-seq) of mouse lungs infected with MRSA. BALB/cCrSlc mice were infected with MRSA ((5.0 × 10^5^ CFU per mouse)) in the presence of *P. int.* sup. or *P. int.* sup. (SOL). At 12 h post-infection, total mRNA was extracted from mouse lungs and analyzed via bulk RNA-seq (*n* = 3). Differentially expressed genes (DEGs) were identified using a threshold of adjusted P < 0.05 and |log_2_ fold change| > 1.0. **(A)** Principal component analysis plot. **(B)** Number of upregulated (red) and downregulated (green) genes in each comparison. **(C)** Volcano plot showing significant DEGs in *P. int.* sup. compared with control medium. **(D)** Volcano plot showing significant DEGs in *P. int.* sup. (SOL) compared with *P. int.* sup. **(E)** Volcano plot showing significant DEGs in *P. int.* sup. (SOL) compared with control medium.

For exploring the macrophage-associated mRNA expression, bulk RNA-seq was performed. In mouse lungs infected with MRSA in the presence of *P. int.* sup. or *P. int.* sup. (SOL) at 12 h post-infection, principal component analysis showed the *P. int.* sup. (SOL) group clustered closely with the control group and distinctly from *P. int.* sup. (**Figures 4A**). The number of differentially expressed genes (DEGs) across all comparisons revealed distinct expression patterns. Between the *P. int*. sup. and control groups, more genes were upregulated (1, 632 genes) than downregulated (1, 207 genes). In contrast, between the *P. int*. sup. (SOL) and *P. int*. sup. groups, more genes were downregulated (1, 373 genes) than upregulated (1, 009 genes). Furthermore, compared to the control group, the *P. int*. sup. (SOL) group had more upregulated (102) than downregulated genes (16) (**Figure 4B**). In a volcano plot comparing the *P. int*. sup. group with the control group, inflammatory genes, including *Tnf*, *Ifng*, *Il1r2*, *Cxcl2*, and *Clec4e*, were upregulated (**Figure 4C**). In contrast, *Il1r2*, *Cxcl2*, and *Clec4e* were downregulated in a comparison between the *P. int*. sup. (SOL) and *P. int*. sup. groups (**Figure 4D**). When comparing the *P. int*. sup. (SOL) and control groups, genes associated with antimicrobial macrophage functions, such as *Clec4e*, *Cybb*, and *Irgm1*, were upregulated (**Figure 4E**).

In the enrichment pathway analysis, the type 1 IFN-related pathway was downregulated, whereas several key immune-related pathways were significantly upregulated in the *P. int.* sup. group. These included pathways associated with phagocytosis, IL-1, type II IFN, and nitric oxide (NO) (**Table 1**). In contrast, in the *P. int*. sup. (SOL) group compared to that in the *P. int*. sup. group, a pathway associated with IFN-β was upregulated, whereas those associated with IL-6, IL-1, phagocytosis, and type II IFNs were downregulated (**Table 2**). Furthermore, in the *P. int*. sup. (SOL) group compared to that in the control medium group, several key pathways associated with type II IFNs, IFN-β, phagocytosis, and IL-1 were significantly upregulated (**Table 3**). Treatment with IFN-β1 or IFN-γ did not improve the survival rate of MRSA-VAP mice exposed to *P. int*. sup. (**Supplementary Figure S2**). Treatment with IFN-β1 or IFN-γ did not improve the survival rate of MRSA-VAP mice exposed to *P. int*. sup. (**Supplementary Figure S2**). In summary, *P. int*. sup. (SOL) were upregulated macrophage-associated mRNA expressions (eg; *Clec4e*, *Cybb*, and *Irgm1), and* pathways associated with type II IFNs, IFN-β, phagocytosis, and IL-1.

**Table 1:** Gene ontology (GO) analysis of bulk RNA-seq data from MRSA-infected mouse lungs: comparison of *P. int*. sup and control groups.

ID	Description	Up/down	p. adjust
GO:0006909	phagocytosis	up	1.97E-11
GO:0070555	response to interleukin-1	up	1.05E-06
GO:0034341	response to type II interferon	up	1.88E-05
GO:0045428	regulation of nitric oxide biosynthetic process	up	3.90E-05
GO:0060338	regulation of type I interferon-mediated signaling pathway	down	0.04061286

Total RNA from BALB/cCrSlc mice infected with MRSA (5.0 × 10^5^ CFU per mouse) for 12 h in the presence of *P. int.* sup. or control medium was analyzed via bulk RNA-seq (*n* = 3). GO analysis of the *P. int.* sup. group compared with the control group was shown.

**Table 2:** GO analysis of bulk RNA-seq data from MRSA-infected mouse lungs: comparison of *P. int*. sup. (SOL) and *P. int.* sup. groups.

ID	Description	Up/down	p. adjust
GO:0035456	response to interferon-beta	up	1.39E-12
GO:0032635	interleukin-6 production	down	2.85E-08
GO:0070555	response to interleukin-1	down	0.00104424
GO:0006909	phagocytosis	down	0.00136306
GO:0034341	response to type II interferon	down	0.01540558

Total RNA from BALB/cCrSlc mice infected with MRSA (5.0 × 10^5^ CFU per mouse) for 12 h in the presence of *P. int.* sup. (SOL) was analyzed via bulk RNA-seq (*n* = 3). GO analysis of the *P. int.* sup. (SOL) group compared with the *P. int.* sup. group was shown.

**Table 3:** GO analysis of bulk RNA-seq data from MRSA-infected mouse lungs: comparison of *P. int*. sup. (SOL) and control groups.

ID	Description	Up/down	p. adjust
GO:0034341	response to type II interferon	up	8.01E-25
GO:0035458	cellular response to interferon-beta	up	7.10E-22
GO:0071222	cellular response to lipopolysaccharide	up	5.38E-14
GO:0006909	phagocytosis	up	1.26E-13
GO:0071347	cellular response to interleukin-1	up	0.00072237

Total RNA from BALB/cCrSlc mice infected with MRSA (5.0 × 10^5^ CFU per mouse) for 12 h in the presence of *P. int.* sup. (SOL) was analyzed via bulk RNA-seq (*n* = 3). GO analysis of the *P. int.* sup. (SOL) group compared with the control group was shown.

### 3.5 SOL pretreatment reduces MRSA burden and upregulates *Tnf-α* mRNA expression in AMLCs infected for 24 h

**Figure 5:**
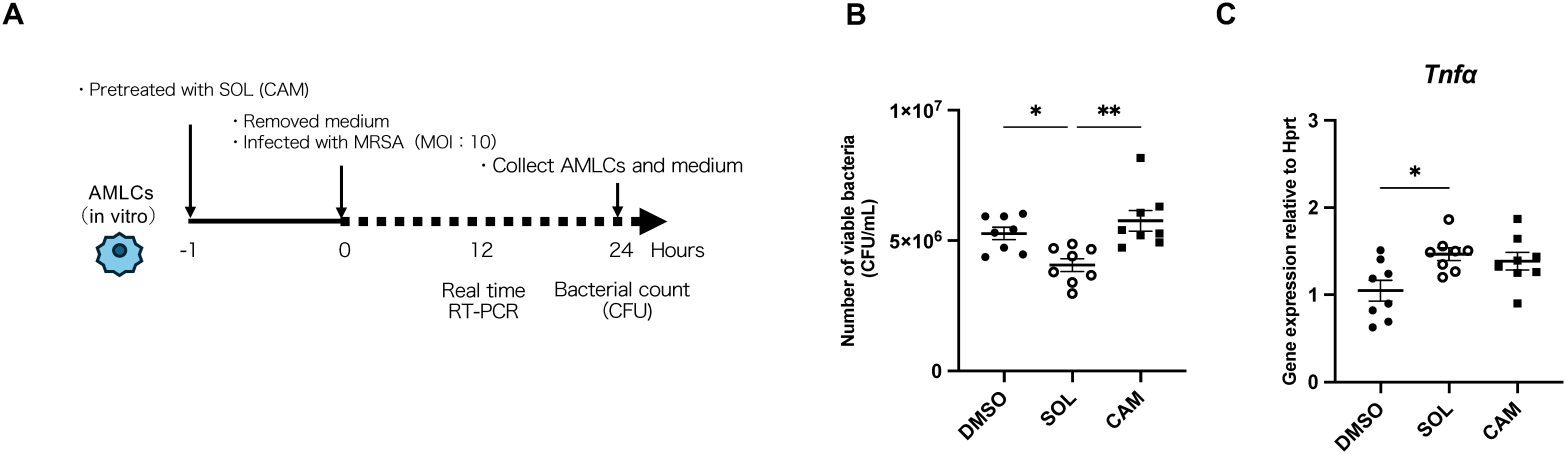
The bactericidal function of mouse alveolar macrophage-like cells (AMLCs) against MRSA and *Tnf-α* expression *in vitro*. **(A)** Experiment outline with the solid line indicating the presence of SOL or CAM, and the dotted line denoting the absence of SOL or CAM. **(B)** Bacterial counts of MRSA pretreated with SOL, CAM, or dimethyl sulfoxide (control) and then incubated with AMLCs (1.0 × 10^5^ cells/well) for 1 h prior to infection. After removal of the medium, cells were incubated with MRSA (1.0 × 10^6^ CFU/well) for 24 **(h)** The number of bacteria was shown (*n* = 8; Kruskal–Wallis test followed by Dunn’s multiple comparison test; *P < 0.05, **P < 0.01). **(C)** mRNA expression of MRSA pretreated with SOL, CAM, or dimethyl sulfoxide (control) and then incubated with AMLCs (1.0 × 10^5^ cells/well) for 1 h prior to infection. After removal of the medium, cells were incubated with MRSA (1.0 × 10^6^ CFU/well) for 12 **(h)***Tnf-α* mRNA expression was shown. (*n* = 7–8; Kruskal–Wallis test followed by Dunn’s multiple comparison test; *P < 0.05). Gene expression levels were normalized to *Hprt* as an internal control. Each experiment was conducted independently in duplicate. Graphs represent cumulative samples; results are expressed as mean ± SEM. The concentration of SOL was 0.00019 μg/mL, and that of CAM was 0.0039 μg/mL.

To assess the effects of SOL on alveolar macrophages, the experiments using AMLCs were conducted *in vitro*. An outline of the experiment is shown in **Figure 5A**. In conditions where AMLCs were pretreated with SOL or CAM 1 h prior to infection, followed by medium removal and incubation with MRSA (multiplicity of infection [MOI]: 10) for 24 h, the bacterial burden was significantly decreased in the SOL groups (**Figure 5B**).

mRNA expression of macrophage genes associated with phagocytosis and bactericidal activity in AMLCs pretreated with SOL or CAM 1 h prior to infection, followed by medium removal and incubation with MRSA (MOI: 10) for 12 h, revealed a significant upregulation of *Tnf-α* expression in the SOL groups compared to that in the other groups (**Figure 5C**). No significant differences were observed in the expression of other genes (**Supplementary Figure S3**).

*P. int.* sup. does not promote the growth of MRSA *in vitro*. Growth curves of MRSA incubated with *P. int.* sup. (both undiluted and diluted) were measured from 0 to 6 h using a plate reader. Optical density at 600 nm did not differ significantly among the groups (**Supplementary Figure S4**).

Treatment with SOL did not promote overall macrophage recruitment but tended to increase the proportion of alveolar macrophages in mouse lungs. Flow cytometry analysis in mouse lungs 24 h post-oropharyngeal administration of SOL (100 μL per mouse) showed no significant differences in macrophages and neutrophils among groups (**Supplementary Figures S5A, B**). In contrast, alveolar macrophages showed a significant increase (**Supplementary Figure S5C**). No significant differences were observed in *Tnf-α* expression in mouse lungs 24 h post-administration with SOL (100 μL (0.019 μg) per mouse) (**Supplementary Figure S6A**) or in AMLCs (1.0 × 10^5 cells/well) incubated with SOL (0.00019 μg/mL) or dimethyl sulfoxide (control) for 12 h (**Supplementary Figure S6B**). In conclusion, SOL reduced the bacterial burden and increased *Tnf-α* expression *in vitro*.

## 4 Discussion

This study demonstrated that *P. int.* sup. exacerbates MRSA-VAP, whereas *P. int.* sup. (SOL), *P. int.* sup. (CAM), or heat-treated *P. int.* sup. mitigate this effect. These findings suggest that the protein components of *P. int*. sup. are likely responsible for the exacerbation of MRSA-VAP. The observed virulence activity is probably driven by heat-sensitive protein components rather than by endotoxins, which are typically lipid-based and generally resistant to heat and protease treatment. Furthermore, regarding the therapeutic mechanism, SOL may inhibit bacterial protein synthesis by targeting the 50S ribosomal subunit of *P. int.*, thereby reducing the production of protein components that worsen MRSA-VAP. Further studies are warranted to identify the specific protein components of *P. int*. sup. that contribute to this effect.

A previous study reported that *P. int.* sup. upregulates α-hemolysin expression (Yamashita et al., 2020). α-hemolysin, a ~30 kDa protein produced by *S*. *aureu*s (Luo et al., 2021), has been implicated in disrupting the capillary barrier and promoting airway bleeding (Berube and Bubeck Wardenburg, 2013). SOL and CAM may inhibit bacterial protein synthesis by targeting the 50S ribosomal subunit of *P. int*., thus reducing α-hemolysin production. Flow cytometric analysis in mouse lungs at 24 h post-infection in the MRSA-VAP model revealed that neutrophils (Ly6G^+^, F4/80^−^) were more abundant in the lungs of the *P. int*. sup. group than those in the control medium group. In contrast, this increase was attenuated in both the *P. int*. sup. (SOL) and *P. int*. sup. (CAM) groups. Conversely, the proportion of macrophages (Ly6G^−^, F4/80^+^) was highest in the *P. int.* sup. (SOL) group and lowest in the *P. int*. sup. group. Neutrophils have been reported to play a central role in the innate immune response against MRSA infection (De Jong et al., 2019), while macrophages have been shown to attenuate neutrophilic inflammation in the lungs (Han et al., 2023). Excessive neutrophilic inflammation, however, can cause tissue damage, thrombosis, and fibrosis. These findings suggest that *P. int*. sup. exacerbates MRSA-VAP by impairing macrophage recruitment and enhancing neutrophilic inflammation—effects that were dampened by treatment with *P. int*. sup. (SOL) or *P. int*. sup. (CAM). Notably, *P. int.* sup. (SOL) not only mitigated the impairment of macrophage recruitment but also promoted macrophage accumulation in the lungs.

Analysis of mRNA expression in mouse lungs 12 h post-infection with MRSA in the presence of each supernatant revealed several notable changes. Expression of *Ly6G*, a neutrophil surface marker (Daley et al., 2008), was significantly upregulated in the *P. int*. sup. group compared to that in the control group. In contrast, expression of *Ccr2*, a chemokine receptor involved in monocyte recruitment (Tsou et al., 2007; Serbina et al., 2012), was significantly reduced in the *P. int*. sup. group compared to that in the control and *P. int.* sup. (SOL) groups. *Marco*, a scavenger receptor involved in pathogen recognition and phagocytosis (Kraal et al., 2000), was also downregulated in the *P. int.* sup. group, whereas its expression was higher in the *P. int*. sup. (SOL) group than in any other group. Additionally, *Ifn-γ* expression was significantly elevated in the *P. int.* sup. (SOL) group compared to that in the *P. int*. sup. (CAM) group. These findings suggest that *P. int*. sup. promotes neutrophilic inflammation by upregulating *Ly6G* expression and impairs macrophage recruitment by downregulating *Ccr2* and *Marco* expression. In contrast, *P. int.* sup. (SOL) and *P. int.* sup. (CAM) attenuated these effects. Furthermore, *P. int.* sup. (SOL) specifically enhanced the expression of *Tnf-α* and *Ifn-γ*, which may contribute to the activation of immune cells, particularly macrophages.

Analysis of bulk RNA-seq of lung tissue from MRSA-VAP mice treated with either *P. int.* sup. or *P. int.* sup. (SOL) revealed distinct transcriptional profiles. *P. int.* sup. upregulated pathways associated with phagocytosis, IL-1, type II IFN, and NO, while downregulating type I IFN signaling. In contrast, *P. int.* sup. (SOL) attenuated these changes. Macrophages, activated by IFN-γ signaling (Kang et al., 2018), subsequently induce the production of proinflammatory cytokines such as IL-1 and IL-6 (Scheibenbogen and Andreesen, 1991), which enhance macrophage bactericidal functions, including phagocytosis (Pidwill et al., 2020) and NO-mediated killing (Richardson, Dunman et al., 2006). These findings suggest that *P. int.* sup. induces excessive activation of T cells, neutrophils, and macrophages, potentially contributing to acute respiratory distress syndrome or hypercytokinemia, whereas *P. int.* sup. (SOL) appropriately attenuates these immune responses. Type I IFNs, including IFN-α and IFN-β, are critical mediators of antiviral immunity (Garcia-Sastre and Biron, 2006). However, previous studies have shown that *Ifnar*-deficient mice are protected from *S. aureus*-associated mortality (Martin et al., 2009). In our study, treatment with IFN-β1 or IFN-γ did not improve the survival rate of MRSA-VAP mice exposed to *P. int*. sup. (**Supplementary Figure S2**), suggesting that these cytokines may not play a central role in survival under these conditions.

Overall, our results indicate that *P. int*. sup. (SOL) suppresses the activation of inflammatory pathway, thereby improving survival, lowering bacterial burden in the lungs, and alleviating histopathological damage in mice. These findings suggest that SOL exerts its therapeutic effects by targeting *P. int.*, consequently reducing the production of proteinaceous components that drive immune overactivation.

Notably, in the *P. int*. sup. (SOL) group, several key pathways associated with macrophage phagocytosis and bactericidal function were significantly upregulated. Furthermore, *in vitro* experiments using AMLCs pretreated with a very low concentration of SOL or CAM for 1 h prior to MRSA infection, followed by medium replacement and 24-h incubation, demonstrated a marked reduction in bacterial load in the SOL group along with a significant upregulation of *Tnf-α* expression. The mRNA levels of genes related to macrophage phagocytosis and bactericidal functions, such as *Marco*, *Cybb*, and *Irgm1*, were upregulated in the SOL group (**Supplementary Figure S3**). Previous studies have shown that macrolides not only exert anti-inflammatory effects in response to lipopolysaccharide stimulation but also directly enhance alveolar macrophage phagocytosis in patients with chronic obstructive pulmonary disease (COPD) (Xu et al., 1996; Yamaryo et al., 2003). Clinically, azithromycin has been reported to increase the phagocytic capacity of alveolar macrophages in patients with COPD (Hodge, Hodge et al., 2006). Additionally, studies using macrophages isolated from patients with cystic fibrosis have shown that macrolides promote *S*. *aureus* phagocytosis (Tarique et al., 2024).

Our findings suggest that even a very low concentration of SOL enhances macrophage phagocytosis and bactericidal activity in AMLCs, possibly through the upregulation of *Tnf-α*. Moreover, growth curve analyses of MRSA cultured with *P. int.* sup. (both undiluted and diluted) revealed no significant differences among the groups (**Supplementary Figure S4**), indicating that *P. int*. sup. does not act directly on MRSA but instead modulates the immune response by influencing macrophages. In the absence of bacterial infection, SOL did not change the total number of macrophages but significantly increased alveolar macrophage numbers in the lungs of mice (**Supplementary Figure S5**). *Tnf-α* mRNA expression was not significantly different among groups *in vivo* and *in vitro* (**Supplementary Figure S6**). These results suggested that SOL might exert preconditioning against respiratory challenge and activate its function primarily under bacterial infection.

This study has several limitations. First, we did not evaluate whether SOL decreases the production of pathogenic factors produced by *P. int*. *in vivo*. Second, oral administration of SOL did not improve MRSA-VAP exacerbated by *P. int.* sup. This may be explained by the possibility that SOL inhibits the production of these pathogenic factors rather than neutralizing them. Third, we could not identify the detailed component in the *P. int*. sup inhibited by SOL, and it is unclear what the effect of the combination of heat-labile and heat-resistant components is. Fourth, we used different mouse strains for *in vitro* and *in vivo* studies. Therefore, it may not be completely consistent with immune responses in both experiments. Fifth, *in vivo* infection model, oropharyngeally administration procedure may not fully recapitulate aerosol exposure in humans.

Future research should assess alternative delivery methods for SOL, such as inhalation or direct intratracheal administration, to determine whether localized delivery can enhance its efficacy in mitigating MRSA-VAP exacerbated by *P. int*. Moreover, further research is warranted to evaluate whether very low concentrations of SOL can augment macrophage phagocytosis and bactericidal activity during infections involving intracellular bacteria, such as those caused by mycobacteria. Regarding identification of pathogenic factors in *P. int*. sup., components should be purified from *P. int*. sup. by using ion-exchange chromatography (IEX) and size-exclusion chromatography (SEC) for polishing. Target-positive fractions were pooled, and then the apparent molecular weight was estimated by SDS–PAGE. The corresponding protein band(s) were subjected to LC–MS/MS for protein identification by database searching with FDR-controlled criteria.

In conclusion, this study demonstrates that *P. int*. sup. exacerbates MRSA-VAP, primarily by overstimulating immune cell responses—particularly neutrophil recruitment—and inducing intrabronchial hemorrhage. In contrast, *P. int*. sup. (SOL) mitigated these harmful effects. This exacerbation appears to be driven by protein components within *P. int*. sup. Additionally, even a very low concentration of SOL may exert therapeutic effects by activating alveolar macrophages *in vitro*, as evidenced by increased expression of immune-related genes, such as Tnf-α. Although this study has several limitations, such as the effect of SOL, especially oral administration *in vivo*, components in the *P. int.* sup inhibited by SOL, and different mouse strains for *in vitro* and *in vivo* studies, these findings highlight a previously unrecognized role for low concentrations of SOL in modulating host immunity and promoting intracellular bacterial clearance. SOL may attract attention to the immune activation therapy in the future.

## Data Availability

The datasets presented in this study can be found in online repositories. The names of the repository/repositories and accession number(s) can be found in the article/**Supplementary Material**.
