# Supplementary material for: Solithromycin mitigates Prevotella intermedia–induced methicillin-resistant Staphylococcus aureus ventilator-associated pneumonia by enhancing alveolar macrophage function
Source: Front Cell Infect Microbiol. 2026 Feb 9;16:1723186. doi: 10.3389/fcimb.2026.1723186 (PMC12926392; doi:10.3389/fcimb.2026.1723186)
Supplement: Supplementary file 1 [file Table1.docx]

Supplementary Material

## Supplementary Figures

**
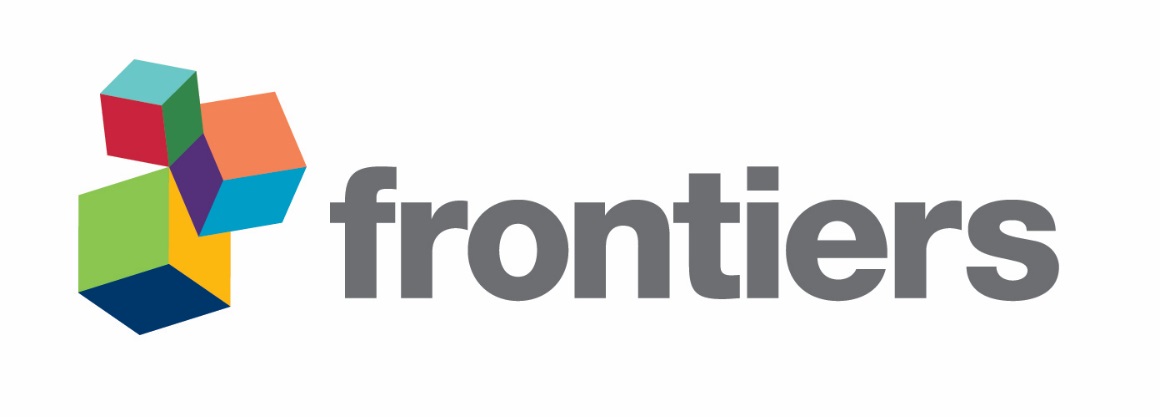
**

**Supplementary Figure 1.** Flow cytometry assay for cell surface antigen on cells in the lung. Cells were gated staining for CD3, CD19, CD4, CD8a, TCRgd and CD161 24 h post-infection, and the frequency of each population was quantified as a percentage of total cells.

1. B cells (CD3 (-) and CD19 (+)): *P. int.* sup. group had similar bacterial counts in the lung as the control group (n = 7-8).
2. CD4+ T cells (CD3 (+) and CD19 (-), CD4 (+) and CD8a (-)): *P. int.* sup. (CAM) The group had similar bacterial counts in the lung as the other groups (n = 7-8).
3. CD8+ T cells (CD3 (+) and CD19 (-), CD4 (-) and CD8a (+)): *P. int.* sup. (CAM) The group had similar bacterial counts in the lung as the other groups (n = 7-8).

(D) γδ T cells (CD3 (+) and CD19 (-) and TCRgd (+)): *P. int.* sup. (CAM) The group had similar bacterial counts in the lung as the other groups (n = 7-8).

(E) NK cells (CD3 (-) and CD19 (-) and CD161 (+)): *P. int.* sup. (CAM) The group had similar bacterial counts in the lung as the other groups (n = 7-8). Each experiment was independently conducted in duplicate. Graphs represent the cumulative samples; results are expressed as mean ± SEM. The Kruskal–Wallis test followed by Dunn’s multiple comparison test was used for statistical analysis.

**Supplementary Figure 2.** The treatment with IFN-β1 or IFN-γ against the exacerbation of MRSA-VAP caused by *P. int.* sup.

(A) Survival of C57BL/6J mice treated with oropharyngeal administration of IFN-β1 (1 μg/mouse) 2 hours prior to infection with MRSA (5.0 × 10^5^ colony-forming unit [CFU] per mouse) in the presence of *P. int.* sup. or control medium (n = 4).

(B) Survival of C57BL/6J mice treated with oropharyngeal administration of IFN-γ (1 μg/mouse) 2 hours prior to infection with MRSA (5.0 × 10^5^ CFU per mouse) in the presence of *P. int.* sup. or control medium (n = 4).

Each experiment was independently conducted once.


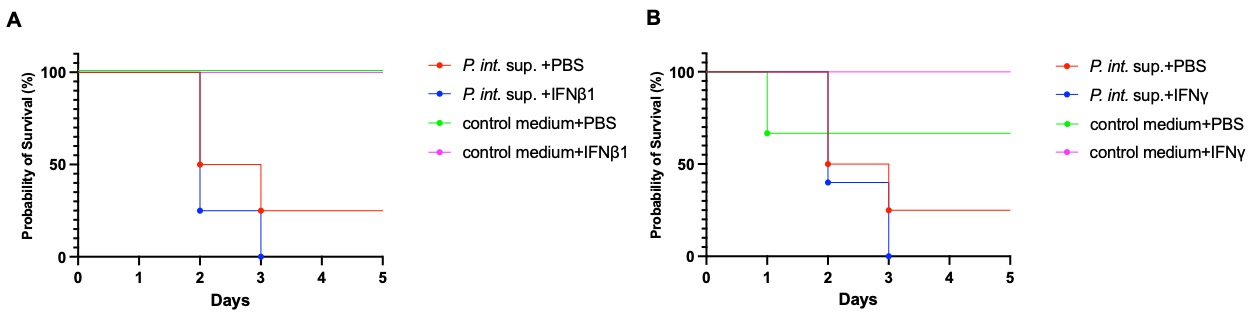


**Supplementary Figure 3.** The mRNA expression of genes associated with macrophage phagocytosis and bactericidal functions *in vitro.*

AMLCs (1.0 × 10^5^/well) were pretreated with SOL or CAM for 1 h prior to infection, and the medium was removed, and then incubated with MRSA (1.0 × 10^6^ CFU/well) for 12 h. Then, AMLCs were collected and assessed for mRNA expression. The concentration of SOL was 0.00019 μg/mL, and that of CAM was 0.0039 μg/mL. (A) *Marco* mRNA expression was shown (n = 8). (B)*Cybb* mRNA expression was shown (n = 8). (C)*Irgm1* mRNA expression was shown (n = 7-8). Each experiment was independently conducted in duplicate. Graphs represent the cumulative samples; results are expressed as mean ± SEM. The Kruskal–Wallis test followed by Dunn’s multiple comparison test was used for statistical analysis


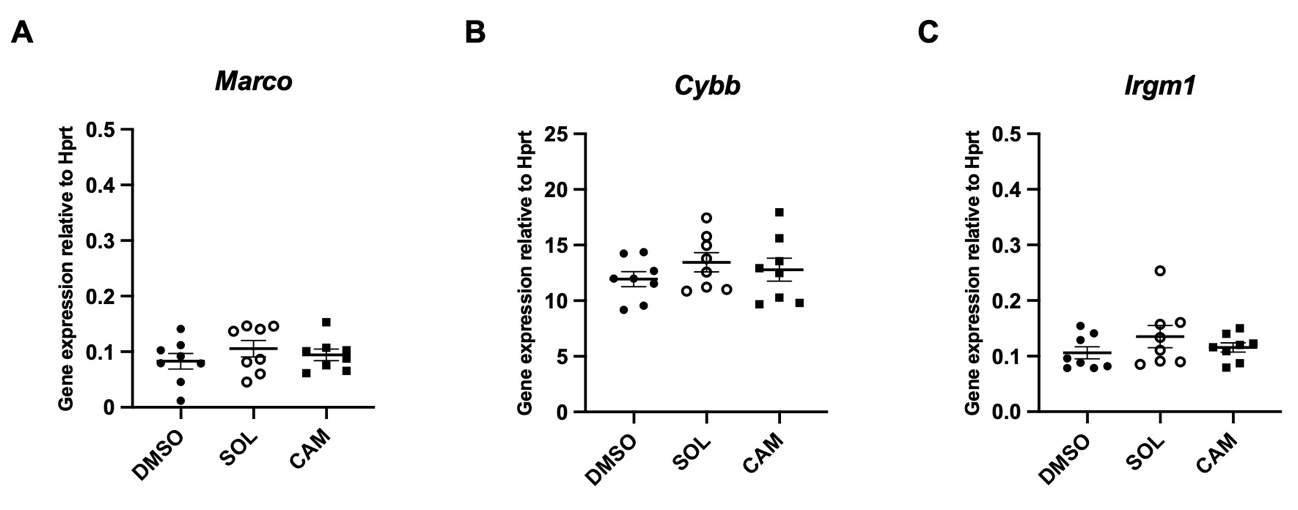
.

**Supplementary Figure 4.** *Prevotella intermedia* culture supernatant (*P. int.* sup.) does not affect the growth curve of MRSA*.*

MRSA was cultured in the presence of *P. int.* sup., *P. int.* sup. (SOL) or *P. int.* sup. (CAM) (undiluted, 2-fold, 3-fold, and 4-fold dilutions) mixed with GAM broth in a 96-well plate. The growth of MRSA was assessed by measuring optical density at 600 nm (OD₆₀₀) daily from 0 hours to 6 hours using a plate reader.


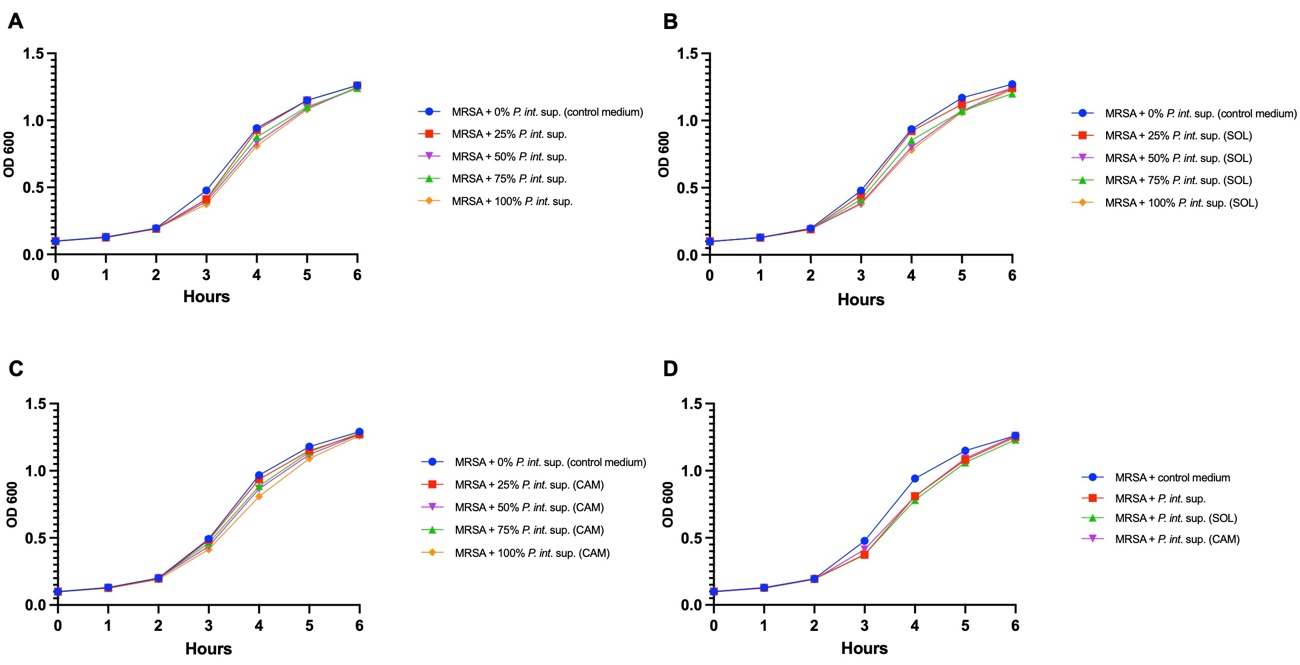


**Supplementary Figure 5.** Flow cytometry analysis of cell surface antigens in mouse lungs 24 hours after oropharyngeal administration of solithromycin (SOL; 100 μL at the specified concentration per mouse) or dimethyl sulfoxide (control). Cells were gated based on Ly6G, F4/80, CD11c, and Siglec-F. The frequency of each population was quantified as a percentage of the total number of analyzed cells. (A) Staining for macrophages (Ly6G−, F4/80+, n = 4; one-way analysis of variance (ANOVA) test followed by Tukey’s multiple comparison test). (B) Staining for neutrophils (Ly6G+, F4/80−, n = 4; one-way analysis of variance (ANOVA) test followed by Tukey’s multiple comparison test). (C) Staining for alveolar macrophages (Ly6G−, F4/80+, CD11c+, Siglec-F+, n=4; one-way ANOVA test followed by Tukey’s multiple comparison test; *P < 0.05). Each representative experiment was repeated.

**Supplementary Figure 6.** *Tnfα* mRNA expression was shown (A) in mouse lungs 24 h post-administration with solithromycin (SOL; 100 μL (0.019 μg) per mouse) or dimethyl sulfoxide (control). (n = 8; Mann–Whitney U-test;). (B) in AMLCs (1.0 × 10^5^ cells/well) incubated with SOL (0.00019 μg/mL) or dimethyl sulfoxide (control) for 12 h. After removal of the medium, cells were collected. Each experiment was independently conducted in duplicate. Graphs represent the cumulative samples; results are expressed as mean ± SEM. The Mann-Whitney U-test was used for statistical analysis.

## Supplementary Tables

**Supplementary Table 1.** **Antibodies used for flow cytometry.**

| Antibody | Manufacturer | Clone | Cat. No. |
| --- | --- | --- | --- |
| anti-F4/80 | BioLegend | BM8 | 123110 |
| anti-SiglecF | BioLegend | 1A8 | 127614 |
| anti-CD45.2 | BioLegend | 104 | 109814 |
| anti-CD11b | BioLegend | M1/70 | 101229 |
| anti-CD11c | BioLegend | N418 | 117343 |
| anti-CD80 | BioLegend | 16-10A1 | 104724 |
| anti-CD163 | BioLegend | S15049I | 155319 |
| anti-CD3 | BioLegend | OKT3 | 317328 |
| anti-CD4 | BioLegend | GK1.5 | 100422 |
| anti-CD8a | BioLegend | 53-6.7 | 100714 |
| anti-CD19 | BioLegend | 1D3/CD19 | 152404 |
| anti-TCRgd | BioLegend | GL3 | 118129 |
| anti-CD161 | BioLegend | PK136 | 108731 |

**Supplementary Table 2. Primers used for real-time RT-PCR.**

| Primer | Manufacturer | Assay ID |
| --- | --- | --- |
| *Ly6g* | Thermo Fisher Scientific | Mm04934123_m1 |
| *Ccr2* | Thermo Fisher Scientific | Mm00438270_m1 |
| *Marco* | Thermo Fisher Scientific | Mm00440265_m1 |
| *Tnfa* | Thermo Fisher Scientific | Mm00443258_m1 |
| *Infg* | Thermo Fisher Scientific | Mm01168134_m1 |
| *Cybb* | Thermo Fisher Scientific | Mm01287743_m1 |
| *Irgm1* | Thermo Fisher Scientific | Mm00492595_m1 |
